# Supplementary material for: Neutralizing Activity of Sera from Sputnik V-Vaccinated People against Variants of Concern (VOC: B.1.1.7, B.1.351, P.1, B.1.617.2, B.1.617.3) and Moscow Endemic SARS-CoV-2 Variants
Source: Vaccines (Basel). 2021 Jul 12;9(7):779. doi: 10.3390/vaccines9070779 (PMC8310330; doi:10.3390/vaccines9070779)
Supplement: Supplementary file 1 [file vaccines-09-00779-s001.zip › vaccines-1278027-supplementary.pdf]

Neutralizing Activity of Sera from Sputnik V Vaccinated People against Variants of Concern (VOC: B.1.1.7, B.1.351, P.1, B.1.617.2, B.1.617.3) and Moscow Endemic SARS-CoV-2 Variants

Vladimir A. Gushchin [1,2,*], Inna V. Dolzhikova [1,*], Alexey M. Shchetinin [1], Alina S. Odintsova [1], Andrei E. Siniavin [1,3], Maria A. Nikiforova [1], Andrei A. Pochtovyi [1], Elena V. Shidlovskaya [1], Nadezhda A. Kuznetsova [1], Olga A. Burgasova [1,4,5], Ludmila V. Kolobukhina [1,4], Anna A. Iliukhina [1], Anna V. Kovyrshina [1], Andrey G. Botikov [1], Aleksandra V. Kuzina [1], Daria M. Grousova [1], Amir I. Tukhvatulin [1], Dmitry V Shcheblyakov [1], Olga V. Zubkova [1], Oksana V. Karpova [4], Olga L. Voronina [1], Natalia N. Ryzhova [1], Ekaterina I. Aksenova [1], Marina S. Kunda [1], Dmitry A. Lioznov [6,7], Daria M. Danilenko [4], Andrey B. Komissarov [7], Artem P. Tkachuck [1], Denis Y. Logunov [1,*], Alexander L. Gintsburg [1,8]

Supplementary

Supplementary methods

Nasopharyngeal swabs collection

The enrollment of patients with a verified diagnosis of COVID-19 and the collection of biological material during the disease progression was conducted at the Moscow City Infectious Disease Clinical Hospital No.1 Moscow Healthcare Department from May to June 2020 (the first wave of the pandemic) and from November 2020 to March 2021 (the second wave of the pandemic). Biomaterial was collected and transported in accordance with the requirements of Sanitary Norms and Regulations of the Russian Federation №1.3.3118-13 and №1.2.036-95. The patients under observation signed informed consent for biological material sampling and underwent survey to gather data for their subsequent use for scientific purposes. The study was approved by the ethics committee (minutes of the Local Ethics Committee No. 2a of May 11, 2020, No. 11a of November 16, 2020, and No. 1 of February 11, 2021).

The material collection was carried out from 1,572 patients (500 subjects during the first wave of the pandemic and 1,072 subjects during the second wave) diagnosed with COVID-19 who were hospitalized at different times from the onset of their first symptoms. The criterion for swab collection was a positive PCR result at the admission to the hospital. Biomaterial (nasopharyngeal swab) was collected from the patients examined by quantitative reverse transcription PCR. SARS-CoV-2 RNA was identified in the samples using a reagent kit for extraction and qualitative determination of SARS-CoV-2 coronavirus RNA using the SARS-CoV-2 FRT RT-PCR method, manufactured by Gamaleya National Research Institute of Epidemiology and Microbiology.

Spike-pseudotyped lentivirus

We have developed a lentivirus-based pseudovirus carrying the GFP reporter gene and S protein of SARS-CoV-2 on the surface of pseudovirus particles. Variants of pseudovirus, carrying the S proteins of the reference Wuhan strain and VOC B.1.1.7 and B.1.351 lineages variants were obtained. In order to evaluate the VNA on a pseudovirus, we have formed a serum panel of volunteers who previously received Sputnik V (table S4 and S5). The serum panel of 8 volunteers receiving two doses of Sputnik V vaccine (table S4) has been tested for neutralizing activity against pseudo-SARS-CoV-2 Wuhan, B.1.1.7 and B.1.351 lineages

Figure S1. **Prevalence of RBD mutations in Russian SARS-CoV-2 variants collected since 2021.** Colored bars represent frequencies of RBD mutations found in Russian SARS-CoV-2 genomes available in GISAID (blue) and the current study (orange) with sample collection dates starting from 2021.

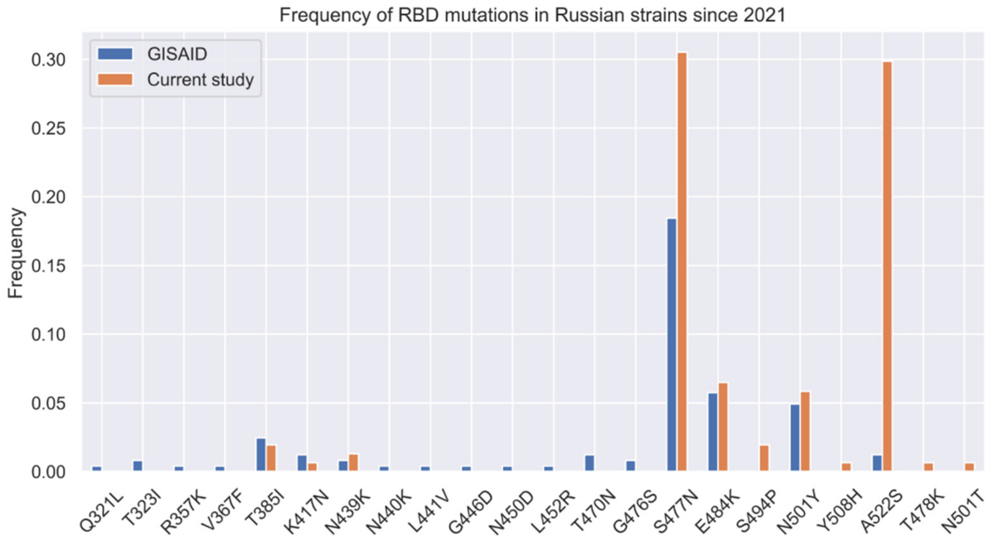

Figure S2. **RBD mutations profiles of the major Russian SARS-CoV-2 genetic lineages**

Colored bars represent RBD mutation frequencies in Russian GISAID sequences for 5 genetic lineages majorly spread in Russia.

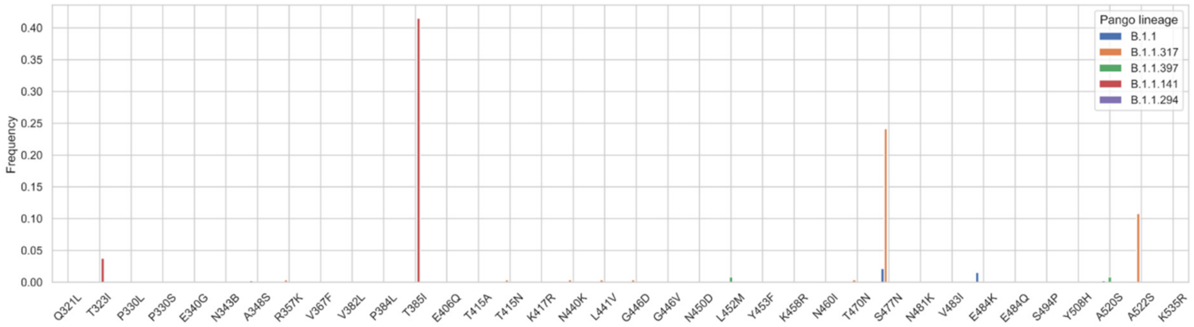

The estimation of variability according to our data in Moscow study shows that mutations S477N and A522S are coupled in 98% of cases (Pearson correlation coefficient 0.99) (figure S3). In contrast, genetic data for Russian variants sequences from GISAID shows that S477N mutation is mostly present separately from A522S.

***Figure S3:* Correlation matrix of co-presence of mutations in the RBD**. Numbers in cells represent the Pearson coefficient for one-hot encoded mutations in RBD according to the data of the current study.

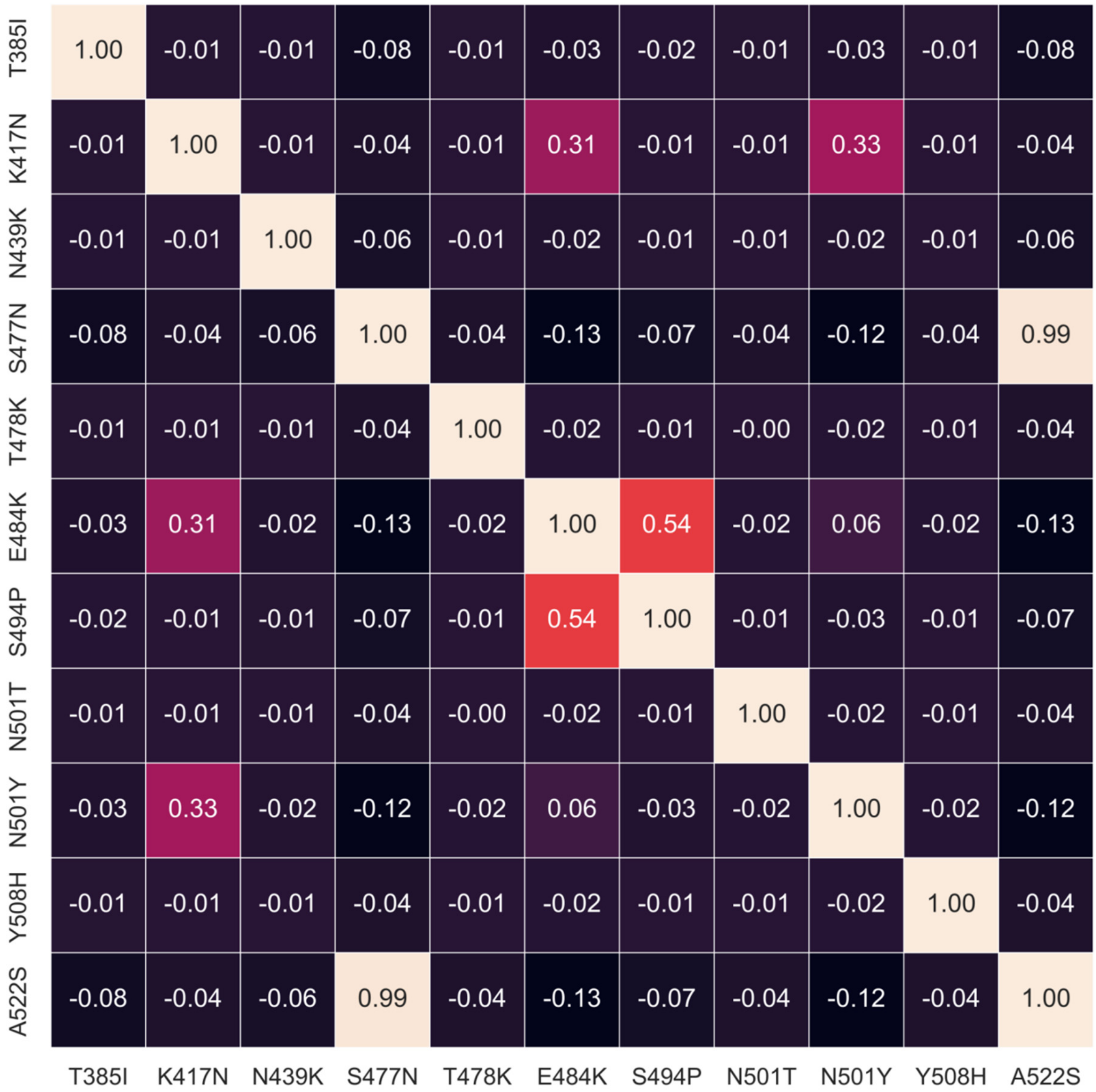

Figure S4: **Neutralization activity of serum samples after two doses of the Sputnik V vaccine recipients against wild-type pseudo-SARS-CoV-2 and B.1.1.7 or B.1.351 spike mutant viruses**. Dose-response curves of neutralizing activity of serum against (A) Wuhan reference strain, (B) lineage B.1.1.7 and (C) lineage B.1.351 SARS-CoV-2 pseudoviruses. Data represent mean value ± SEM of n=3 technical replicates. The numbers (003-013) indicate individual sera.

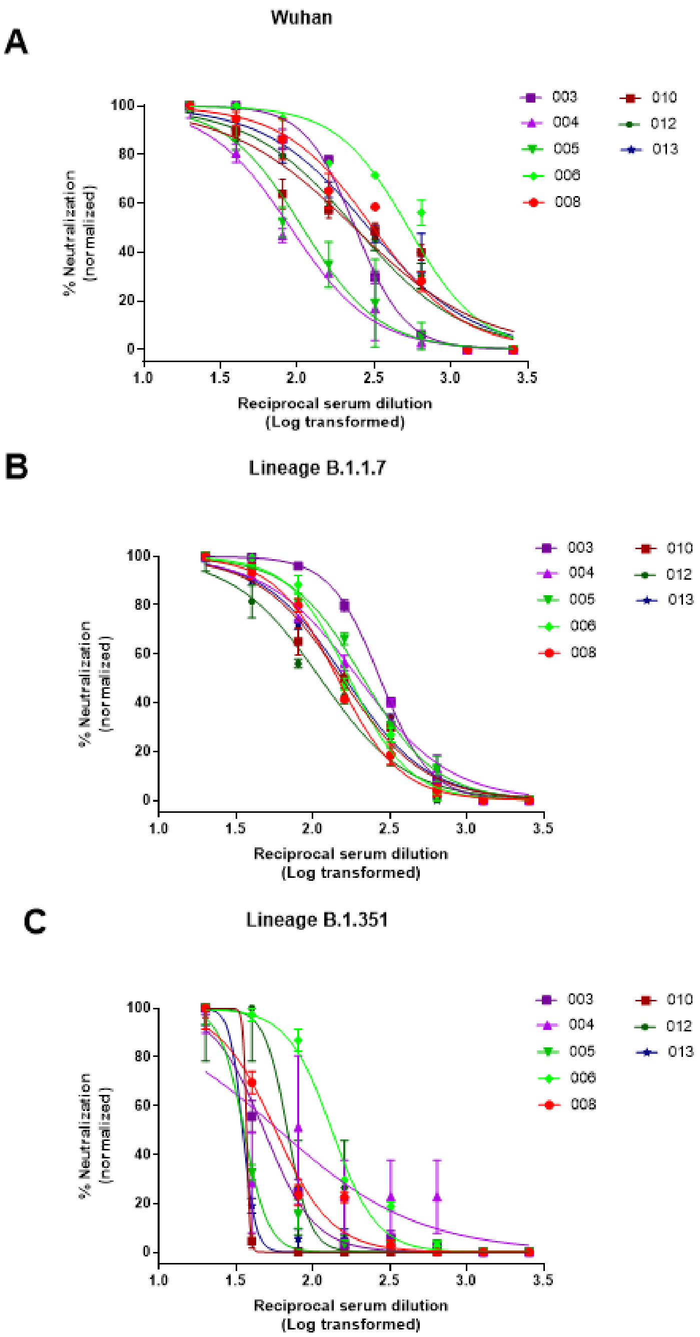

Table S1: **The most common mutations in the RBD as reported in this study**

Position in Spike	385	417	439	477	478	484	494	501	508	522	Amount of appearance in current research (Nov-Mar 2021)	
Wild type	T	K	N	S	T	E	S	N	Y	A	94	55,62%
S477N + A522S	·	·	·	N	·	·	·	·	·	S	47	27,81%
N501Y	·	·	·	·	·	·	·	Y	·	·	8	4,73%
E484K	·	·	·	·	·	K	·	·	·	·	6	3,55%
T385I	I	·	·	·	·	·	·	·	·	·	4	2,37%
E484K + S494P	·	·	·	·	·	K	P	·	·	·	3	1,77%
N439K	·	·	K	·	·	·	·	·	·	·	2	1,18%
K417N + E484K + N501Y	·	N	·	·	·	K	·	Y	·	·	1	0,59%
S477N	·	·	·	N	·	·	·	·	·	·	1	0,59%
T478K	·	·	·	·	K	·	·	·	·	·	1	0,59%
N501T	·	·	·	·	·	·	·	T	·	·	1	0,59%
Y508H	·	·	·	·	·	·	·	·	H	·	1	0,59%

Table S2: Putative phenotypic effect of dominating RBD mutations

Mtation	Increased binding affinity to hACE2 receptor	Escape from some mAbs	Escape some convalescent/vaccinated sera	Increased transmissi-bility	More severe illness	References
T385I	✓					1
K417N	✓	✓				1, 15
K417N+ +E484K+ +N501Y	✓	✓	✓	✓		8, 9, 10, 15
N439K	✓	✓	✓	✓		1, 4
S477N	✓Slightly					1, 13
T478K	✓In silico prediction	✓				11, 12
E484K	✓	✓	✓			1, 2, 9
S494P	✓	✓				16, 17
N501Y	✓	✓Not critical	✓Not critical	✓	✓	1, 3, 5, 6, 7, 10
N501T	✓					1
Y508H		✓				14
A522S	✓					1

Table S3: Characteristics of volunteers' sera used in the study of VNA in a pseudovirus model

**Sera ID**	**Days from the 1st dose**	**Days from the 2nd dose**	**COI*** $_{RBD}$	**COI** $_{Nc}$	**Sex**	**Age**
**003**	147	108	10.8	0.3	F	30-35
**004**	64	44	6.4	0.4	F	25-30
**005**	64	45	9.3	0.5	F	35-40
**006**	136	108	9.1	0.4	F	30-35
**008**	64	45	9.6	0.3	F	35-40
**010**	69	50	9.6	0.3	F	25-30
**012**	70	51	8.2	0.3	F	25-30
**013**	70	50	10.2	0.3	F	25-30

* **COI** - cut-off index

Table S4: **The 50% serum neutralization titres against SARS-CoV-2 pseudoviruses**

Sample name	NT_{50}			% NT_{50}		
	Wuhan	Lineage B.1.1.7	Lineage B.1.351	Wuhan	Lineage B.1.1.7	Lineage B.1.351
**003**	237,3	272,3	47,95	100	114,75	20,21
**004**	89,63	197,5	59,94	100	220,35	66,87
**005**	110,6	211,7	35,47	100	191,41	32,07
**006**	526,8	172	132,4	100	32,65	25,13
**008**	310,4	144,2	56,87	100	46,46	18,32
**010**	234,5	152,9	36,97	100	65,20	15,77
**012**	239	111,4	68,15	100	46,61	28,51
**013**	289,6	161,3	34,83	100	55,70	12,03

The median 50% neutralization titers (NT50) of serum against the pseudo-virus SARS-CoV-2 Wuhan was 1/238, while the median NT50 for the pseudo-SARS-CoV-2 lineage of B.1.1.7 was 1/166. According to the one-way ANOVA test with Dunn's multiple comparisons, these differences were statistically insignificant. In testing the neutralizing activity of the serum against the SA variant of the pseudo-virus of the B.1.351 lineage a significant decrease in NT50 was observed compared to the variants of Wuhan and B.1.1.7 lineage (p<0.05). The average NT50 for B.1.351 was 1/52, this is 75% lower than the value for the reference strain Wuhan.

Supplementary references

1. Rui Wang, Jiahui Chen, Kaifu Gao, Guo-Wei Wei. Vaccine-escape and fast-growing mutations in the United Kingdom, the United States, Singapore, Spain, South Africa, and other COVID-19-devastated countries. arXiv.org https://arxiv.org/abs/2103.08023v2.
2. Xie X, Liu Y, Liu J, et al. Neutralization of SARS-CoV-2 spike 69/70 deletion, E484K and N501Y variants by BNT162b2 vaccine-elicited sera. Nat Med 2021; 27: 620–1.
3. Volz E, Mishra S, Chand M, et al. Transmission of SARS-CoV-2 Lineage B.1.1.7 in England: Insights from linking epidemiological and genetic data. DOI:10.1101/2020.12.30.20249034.
4. Thomson EC, Rosen LE, Shepherd JG, et al. Circulating SARS-CoV-2 spike N439K variants maintain fitness while evading antibody-mediated immunity. Cell. 2021 Mar 4;184(5):1171-1187.e20. doi: 10.1016/j.cell.2021.01.037. Epub 2021 Jan 28. PMID: 33621484; PMCID: PMC7843029.
5. Davies NG, Jarvis CI, CMMID COVID-19 Working Group, et al. Increased mortality in community-tested cases of SARS-CoV-2 lineage B.1.1.7. Nature 2021; published online March 15. DOI:10.1038/s41586-021-03426-1.
6. Muik A, Wallisch A-K, Sänger B, et al. Neutralization of SARS-CoV-2 lineage B.1.1.7 pseudovirus by BNT162b2 vaccine-elicited human sera. Science 2021; 371: 1152–3.
7. Shen X, Tang H, McDanal C, et al. SARS-CoV-2 variant B.1.1.7 is susceptible to neutralizing antibodies elicited by ancestral spike vaccines. Cell Host Microbe 2021; 29: 529–39.e3.
8. Tegally H, Wilkinson E, Giovanetti M, et al. Detection of a SARS-CoV-2 variant of concern in South Africa. Nature 2021; published online March 9. DOI:10.1038/s41586-021-03402-9.
9. Zhou D, Dejnirattisai W, Supasa P, et al. Evidence of escape of SARS-CoV-2 variant B.1.351 from natural and vaccine-induced sera. Cell 2021; published online Feb 23. DOI:10.1016/j.cell.2021.02.037.
10. Wang P., Liu L., Iketani S., Luo Y., Guo Y., Wang M., Yu J., Zhang B., Kwong P.D., Graham B.S. Increased Resistance of SARS-CoV-2 Variants B.1.351 and B.1.1.7 to Antibody Neutralization. bioRxiv. 2021 doi: 10.1101/2021.01.25.428137
11. Rezaei S., Sefidbakht Y., Uskoković V. Comparative molecular dynamics study of the receptor-binding domains in SARS-CoV-2 and SARS-CoV and the effects of mutations on the binding affinity. Journal of Biomolecular Structure and Dynamics; published online 17 Dec 2020. https://doi.org/10.1080/07391102.2020.1860829
12. Muecksch F., Weisblum Y., Barnes C.O., et al. Development of potency, breadth and resilience to viral escape mutations in SARS-CoV-2 neutralizing antibodies. bioRxiv 2021 Mar 8;2021.03.07.434227. doi: 10.1101/2021.03.07.434227. Preprint
13. Starr T.N., Greaney A.J., Hilton S.K., et al. Deep Mutational Scanning of SARS-CoV-2 Receptor Binding Domain Reveals Constraints on Folding and ACE2 Binding. Cell. 2020 Sep 3;182(5):1295-1310.e20. doi: 10.1016/j.cell.2020.08.012. Epub 2020 Aug 11.
14. Li Q., Wu J., Nie J., et al. The Impact of Mutations in SARS-CoV-2 Spike on Viral Infectivity and Antigenicity. Cell. 2020 Sep 3; 182(5): 1284–1294.e9. Published online 2020 Jul 17. doi: 10.1016/j.cell.2020.07.012
15. Fratev F. The N501Y and K417N mutations in the spike protein of SARS-CoV-2 alter

the interactions with both hACE2 and human derived antibody: A Free energy of perturbation study. bioRxiv. 2020; published online Dec 26. DOI:10.1101/2020.12.23.424283.

16. Chakraborty S. Evolutionary and structural analysis elucidates mutations on SARS-CoV2 spike protein with altered human ACE2 binding affinity. Biochem Biophys Res Commun. 2021 Jan 1; 534: 374–380. Published online 2020 Nov 28. doi: 10.1016/j.bbrc.2020.11.075
17. Weisblum Y, Schmidt F, Zhang F, et al. Escape from neutralizing antibodies by SARS-CoV-2 spike protein variants. Elife 2020; 9. DOI:10.7554/eLife.61312.
